# Motion of an Elastic Capsule in a Trapezoidal Microchannel under Stokes Flow Conditions

**DOI:** 10.3390/polym12051144

**Published:** 2020-05-17

**Authors:** Abdollah Koolivand, Panagiotis Dimitrakopoulos

**Affiliations:** Department of Chemical and Biomolecular Engineering, University of Maryland, College Park, MD 20742, USA

**Keywords:** microfluidic, polymer, Lab-on-a-chip, capsule, soft matter, computational modeling, particle sorting, fluid dynamics, membrane deformation

## Abstract

Even though the research interest in the last decades has been mainly focused on the capsule dynamics in cylindrical or rectangular ducts, channels with asymmetric cross-sections may also be desirable especially for capsule migration and sorting. Therefore, in the present study we investigate computationally the motion of an elastic spherical capsule in an isosceles trapezoidal microchannel at low and moderate flow rates under the Stokes regime. The steady-state capsule location is quite close to the location where the single-phase velocity of the surrounding fluid is maximized. Owing to the asymmetry of the trapezoidal channel, the capsule’s steady-state shape is asymmetric while its membrane slowly tank-treads. In addition, our investigation reveals that tall trapezoidal channels with low base ratios produce significant off-center migration for large capsules compared to that for smaller capsules for a given channel length. Thus, we propose a microdevice for the sorting of artificial and physiological capsules based on their size, by utilizing tall trapezoidal microchannels with low base ratios. The proposed sorting microdevice can be readily produced via glass fabrication or as a microfluidic device via micromilling, while the required flow conditions do not cause membrane rupture.

## 1. Introduction

The study of the interfacial dynamics of artificial and physiological capsules in microfluidic channels and blood microvessels provides useful information on the utilization of these particles in chemical, pharmaceutical and physiological processes. For example, understanding the stability of soft particle shapes provides helpful insight on the hydrodynamic aggregation and the effective viscosity of suspensions [1]. Some common approaches for fabrication of artificial capsules such as polymer shell microcapsules are polymer precipitation by phase separation, poly-condensation interfacial polymerization, and interfacial polymerization on the surface of flowing droplets in poly-dimethylsiloxane (PDMS) microfluidic flow-focusing devices [2,3,4]. The deformation of artificial capsules in microchannels is directly associated with drug delivery, soft particle sorting and cell characterization [5,6]. Furthermore, the deformability of red blood cells plays a pivotal role in the oxygen and carbon dioxide exchange between the microcirculation and the body tissues [7], and helps identifying the effects of blood disorders and diseases [8,9,10].

The dynamics of artificial capsules and biological cells in solid ducts is determined by the nonlinear coupling of the deforming hydrodynamic forces with the restoring interfacial forces of the particle membrane. Since the hydrodynamic forces depend strongly on the duct geometry, the force interplay suggests that the deformation, migration and steady-state properties of capsules may be quite different in different types of solid ducts, and also quite different than the properties of other particles (such as solid particles, droplets or vesicles) which develop different interfacial forces [1,11,12,13].

During the last decades the research interest has been mainly focused on the capsule dynamics in cylindrical or rectangular channels, e.g., [12,14,15,16,17]. However, channels with asymmetric cross-sections may also be desirable especially for the lateral migration of capsules which plays a significant role in industrial and physiological processes involving drug delivery, cell sorting and cell characterization [18,19,20].

The lateral migration of capsules has been commonly studied near plane walls or in microchannels with symmetric cross-sections such as cylindrical or rectangular ducts [21,22,23,24,25,26]. By contrast, microchannels with various cross-sections have been employed for the migration and separation of solid particles and cells. Examples include channels with semi-circular, triangular and trapezoidal cross-sections [27,28,29,30,31].

In the present study, we investigate computationally the motion of an elastic spherical capsule in an isosceles trapezoidal microchannel at low and moderate flow rates under the Stokes regime. We focus our interest on the cross-streamline migration of the capsules as well as their steady-state location and shape. We consider the effects of capsule’s initial position and size as well as the channel’s height and base ratio.

A capsule placed along the symmetry plane in the trapezoidal microchannel away from its steady-state location undergoes a cross-streamline migration which is accompanied with a slow tank-treading motion and a deformation decrease. These features are reduced as the capsule approaches its steady-state location near the location where the single-phase velocity of the surrounding fluid is maximized. Owing to the asymmetry of the trapezoidal channel, the capsule’s steady-state shape is asymmetric while its membrane slowly tank-treads. In addition, our investigation reveals that tall trapezoidal channels with low base ratios produce significant off-center migration for large capsules compared to that for smaller capsules for a given channel length.

The integration of mechanistic models and computational techniques allows to simulate and control physical and chemical processes (e.g., suspension polymerization [32]), perform system identification and risk analysis (e.g., pharmaceutical manufacturing [33]), and conduct process and product design. Based on our computational findings, here we pursue the process design objective and propose a new microdevice for the sorting of artificial (e.g., polymer shell capsules) and physiological capsules (e.g., biological cells) based on their size, by utilizing tall trapezoidal microchannels with low base ratios. The proposed sorting microdevice can be readily fabricated via commonly available materials (e.g., glass, PDMS) and used, while the required flow rates do not cause membrane rupture. In addition, the simple design of the proposed microdevice, paves the road for scale out and commercialization. To the best of our knowledge, this is the first computational study that considers the dynamics of elastic capsules in a trapezoidal channel.

## 2. Problem Description

### 2.1. Fluid Dynamics

**Figure 1:**
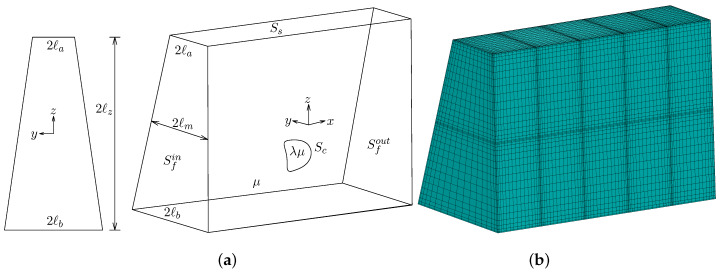
Illustration of (**a**) an isosceles trapezoidal channel with a flowing elastic capsule, and (**b**) a spectral boundary element discretization of the channel.

In this work, we investigate the dynamics of a fluid volume enclosed by a thin elastic membrane, i.e., a three-dimensional capsule with a spherical undisturbed shape, flowing in a trapezoidal microchannel as illustrated in Figure 1a. In addition, we imagine the channel as horizontal and thus the *x*, *z* and *y*-directions will be referred to as length, height and width, respectively. Thus, seeing the capsule from the −y, *z* or *x*-axis represents the front, top or side view, respectively. The cross-section of the channel is an isosceles trapezium with a height $2\ell_{z}$ and a width which varies from $2\ell_{b}$ at the bottom to $2\ell_{a}$ at the top (where $\ell_{b}\geq \ell_{a}$), as shown in Figure 1a. The origin of the co-ordinate system is placed at the symmetry plane of the channel at the middle of the channel height. At the channel centerline (z=0) the median width is $\ell_{m}=\left(\ell_{a}+\ell_{b}\right)/2$.

Both the capsule’s surrounding (fluid 2) and interior (fluid 1) are Newtonian fluids, with viscosities λμ and μ, and the same density. The capsule volume is *V* and thus the capsule size is specified by the radius *a* of a sphere of volume $V=4\pi a^{3}/3$ which is comparable but smaller to the channel half-width $\ell_{b}$.

Under low-Reynolds-number flows, the governing equations in fluid 2 are the Stokes equations and continuity,

(1) $$\nabla \cdot \sigma \equiv -\nabla p+\mu \nabla^{2}u=0\;\;\;\mathrm{and}\;\;\;\;\nabla \cdot u=0$$

where σ is the stress tensor and ***u*** the fluid velocity. Inside the capsule, the same equations apply with the viscosity replaced by λμ.

For the present investigation, the system surface $S_{B}$ consists of the capsule membrane $S_{c}$, the channel’s solid surface $S_{s}$, and the fluid surface $S_{f}$ of the channel’s inlet and outlet far from the capsule. At the capsule membrane, the velocity is continuous and we define the interfacial stress vector (or hydrostatic traction) Δf from the stress tensor σ and the surface unit normal ***n***, i.e.

(2) $$\begin{array}{c} u_{1}=u_{2}=u\;\;\;\mathrm{and}\;\;\;\;\Delta f\equiv n\cdot \left(\sigma_{2}-\sigma_{1}\right) \end{array}$$

Note that the subscripts designate quantities evaluated in fluids 1 and 2, respectively, while ***n*** is the unit normal vector which points into fluid 2. The boundary conditions on the rest surfaces are

(3) $$u=0\;\;\;\mathrm{on}\;\mathrm{the}\;\mathrm{solid}\;\mathrm{boundary}\;S_{s}$$

(4) $$u=u^{\infty }\;\;\;\mathrm{or}\;\;\;\;f=f^{\infty }\;\;\;\mathrm{on}\;\mathrm{the}\;\mathrm{fluid}\;\mathrm{boundary}\;S_{f}$$

where $u^{\infty }$ and $f^{\infty }$ are the undisturbed velocity and force, respectively, at the channel exits far from the capsule.

Based on standard boundary integral formulation, the velocity at a point $x_{0}$ on the system surface $S_{B}$ may be expressed as a surface integral of the force vector f=n·σ and the velocity ***u*** over all points ***x*** on the boundary $S_{B}$,

(5) $$\begin{array}{cc} \Omega \;u(x_{0})= & -\int_{S_{c}}\left[S\cdot \Delta f-\mu (1-\lambda )\;T\cdot u\cdot n\right]\left(x\right)\;dS\\ & -\int_{S_{s}\cup S_{f}}\left(S\cdot f-\mu \;T\cdot u\cdot n\right)\left(x\right)\;dS \end{array}$$

where the coefficient Ω takes values 4πμ(1+λ) and 4πμ for points $x_{0}$ on the surfaces $S_{c}$ and $S_{s}\cup S_{f}$, respectively. The tensors ***S*** and ***T*** are the fundamental solutions for the velocity and stress for the three-dimensional Stokes equations, i.e., known functions of the system surface $S_{B}$ [15,34,35].

Owing to the no-slip condition at the interface, the time evolution of the material points of the membrane is determined via the kinematic condition at the interface

(6) $$\frac{\partial x}{\partial t}=u$$

To determine the capsule motion in the trapezoidal channel, the undisturbed velocity $u^{\infty }$ or force $f^{\infty }$ is required. While we can determine them analytically or numerically from the single-phase flow, it is more efficient to utilize a periodic formulation with sufficiently large periodic length $L_{p}$.

For this purpose, periodic boundary conditions are applied between the inlet and outlet fluid boundaries, $S_{f}^{in}$ and $S_{f}^{out}$, of the channel,

(7) $$u^{in}=u^{out}\;\;\;\mathrm{and}\;\;\;\;f^{in}=-f^{out}-\beta$$

where the superscripts “in” and “out” stand for the inlet and outlet of the channel. The force difference is β=(Δp,0,0) where Δp is the pressure difference between the inlet and outlet of the channel over the length $L_{p}$. Note that in the force condition of Equation (7), the signs result from the choice of the unit normal ***n*** to point into fluid 2, i.e., inside the microchannel.

The flow problem can be solved (i) by assuming a constant average velocity U in the channel and solve for the corresponding pressure difference Δp, or (ii) by assuming a constant pressure difference Δp along a channel length $L_{p}$, solve for the outlets velocity ***u*** and then calculate U. In (i), the known average velocity U constitutes the additional boundary condition required to solve for the additional unknown Δp.

In this work, we utilize case (i), and thus we assume a constant flow rate *Q* in the microchannel. Our computations show that the capsule dynamics is not affected by the periodic length of the channel for $L_{p}>5\ell_{b}$, and thus under this condition, the capsule dynamics is identical to that for the non-periodic problem.

We emphasize that we have validated our computational algorithm for periodic conditions by comparing our findings for droplets with earlier experimental and computational studies for droplet motion in solid conduits under periodic conditions, e.g., [36,37,38].

### 2.2. Membrane Dynamics

We consider an elastic capsule made of a thin strain-hardening membrane following the Skalak et al. constitutive law [39] (and thus called Skalak capsule in this paper) with comparable shearing and area-dilatation resistance but negligible bending resistance. Thus the membrane resistance is described by the (surface) shear and area-dilatation moduli, $G_{s}$ and $G_{a}$, respectively.

In particular, the in-plane tension tensor τ is described by the strain-hardening constitutive law of Skalak et al. [39] which relates τ’s eigenvalues (or principal elastic tensions $\tau_{\beta }^{P},\beta =1,2$) with the principal stretch ratios $\lambda_{\beta }$ by

(8) $$\tau_{1}^{P}=\frac{G_{s}\lambda_{1}}{\lambda_{2}}\left\{\lambda_{1}^{2}-1+C\lambda_{2}^{2}\left[{\left(\lambda_{1}\lambda_{2}\right)}^{2}-1\right]\right\}$$

Note that the reference shape of the elastic tensions is the spherical quiescent shape of the capsule while to calculate $\tau_{2}^{P}$ reverse the $\lambda_{\beta }$ subscripts [39,40].

In addition, we consider that the capsule is slightly over-inflated owing to fabrication. To quantify the capsule over-inflation, we define the prestress parameter $\alpha_{p}$ such that all lengths in the undeformed capsule would be scaled by $\left(1+\alpha_{p}\right)$ relatively to the reference shape [12,15,41]. As a result, the capsule membrane is initially prestressed by an isotropic elastic tension $\tau_{0}=\tau_{\beta }^{P}\left(t=0\right)$ which depends on the employed constitutive law and its parameters but not on the capsule size. For example, for a Skalak capsule with C=1 and $\alpha_{p}=0.05$, the initial membrane tension owing to prestress is $\tau_{0}/G_{s}\approx 0.3401$. In addition, incorporation of prestress into our elastic membrane model removes the buckling instability observed in axisymmetric-like flows (See Section 2 in Ref. [15].)

Based on the membrane description considered in this work, the interfacial stress vector Δf required in Equation (5) is determined by the in-plane tensions, which in contravariant form results into

(9) $$\Delta f=-\nabla_{s}\cdot \tau =-\left(\tau^{\alpha \beta }{|}_{\alpha }\;t_{\beta }+b_{\alpha \beta }\;\tau^{\alpha \beta }\;n\right)$$

where the Greek indices range over 1 and 2, while Einstein notation is employed for (every two) repeated indices. In this equation, the $\tau^{\alpha \beta }{|}_{\alpha }$ notation denotes covariant differentiation, $t_{\beta }=\partial x/\partial \theta^{\beta }$ are the tangent vectors on the capsule surface described with arbitrary curvilinear coordinates $\theta^{\beta }$, and $b_{\alpha \beta }$ is the surface curvature tensor [42,43,44].

This modeling has been proven to represent well artificial and biological capsules with thin membranes where the membrane tensions increase superlinearly with the applied strain [40,44,45]. The strength of the membrane’s strain-hardening nature is described by the dimensionless membrane hardness *C*, which is associated with the scaled area-dilatation modulus $G_{a}$ of the membrane, $G_{a}/G_{s}=1+2C$ [39,40].

We emphasize that real capsules with thin membranes have a small bending resistance resulting from the membrane thickness. The theory of thin shells predicts that for an isotropic membrane, the bending modulus $K_{b}$ is proportional to the (surface) shear modulus $G_{s}$ and the square of the membrane thickness *h*, i.e., $K_{b}∼G_{s}\;h^{2}$, and thus the scaled modulus is $K_{b}/\left(G_{s}\;a^{2}\right)∼{\left(h/a\right)}^{2}$ [46]. For a typical relative thickness of $h/a=10^{-2}$, the scaled modulus is $K_{b}/\left(G_{s}\;a^{2}\right)∼10^{-4}$, and thus the bending resistance is insignificant compared to the shearing and area-dilatation resistance for the overall capsule dynamics.

### 2.3. Computational Methodology

We start our computations with the undisturbed spherical capsule located along the vertical symmetry plane of the trapezoidal channel so that its centroid is $x_{c}=\left(0,0,z_{c}^{0}\right)$. At time t=0 the flow is turned on with a constant flow rate *Q* and average velocity $U=Q/(2\ell_{z}\left(\ell_{a}+\ell_{b}\right))$. The half-width $\ell_{b}$ at the channel bottom is used as the length scale, the velocity scale is U, the pressure is scaled with $\Pi =\mu \;U/\ell_{b}$ and the time scale is $t_{f}=\ell_{b}/U$. The flow is incoming from the left side of the channel and exiting from the right side. We assume that the Reynolds number is small for both the surrounding and the inner flows, and thus the capsule deformation and motion occurs in the Stokes regime.

The numerical solution of the interfacial Stokes flow problem is achieved through our membrane spectral boundary element method written in FORTRAN and parallelized on shared-memory multiprocessor computers via Open MP directives and highly optimized parallel routines from the LAPACK system library [44,47,48]. Our convergence runs for this study covering the entire interfacial evolution show that our results for the interfacial shape are accurate to 2 or 3 significant digits.

The present problem depends on four physical dimensionless parameters, i.e. the capillary number $\mathrm{Ca}=\mu \;U/G_{s}$, the membrane hardness *C*, the prestress $\alpha_{p}$ and the viscosity ratio λ. It also depends on four geometric dimensionless parameters, i.e., the capsule size $a/\ell_{b}$, the channel height $\ell_{z}/\ell_{b}$, the channel’s base ratio $\ell_{a}/\ell_{b}$, and the capsule’s initial position $z_{c}^{0}/\ell_{b}$. Note that the effects of the physical parameters have been studied in our earlier publications on the dynamics of spherical capsules in square and rectangular channels [12,15].

Thus, in this work we restrict our interest in studying the effects of the four geometric dimensionless parameters which represent the most significant problem parameters at low flow rates. In particular, we investigate the dynamics of elastic capsules with membrane hardness C=1 and prestress $\alpha_{p}=0.05$, in flows with capillary number Ca=0.1. Although we present our results for viscosity ratio λ=5, we have also investigated other viscosity ratios (namely, λ=0.1,1) as we report at the end of Section 3.2. We consider capsules smaller than the channel width with size $a/\ell_{b}$ in [0.1,0.6] and varied initial position $z_{c}^{0}/\ell_{b}$, and investigate a wide range of the channel’s height $\ell_{z}/\ell_{b}=1$–4 and base ratio $\ell_{a}/\ell_{b}=0$–1. We focus our interest at the translation and migration of the capsule as well as its steady-state shape and position in the trapezoidal channel.

We emphasize that in this section we presented a detailed description of the physical problem and our mathematical methodology under periodic conditions for the better understanding of the present study but also for future reference. More details on our spectral boundary algorithm and our studies on capsule dynamics in microfluidic flows can be found in our recent publications, e.g., [15,17,41,44,48].

## 3. Results

### 3.1. Capsule Migration from Different Initial Positions

We begin our investigation by considering the dynamics of a capsule with size $a/\ell_{b}=0.55$ left to flow from its quiescent spherical shape at different initial positions $z_{c}^{0}$ on the vertical symmetry plane (i.e. $x_{c}^{0}=y_{c}^{0}=0$) of a trapezoidal channel with height $\ell_{z}/\ell_{b}=2$ and base ratio $\ell_{a}/\ell_{b}=0.25$.

**Figure 2:**
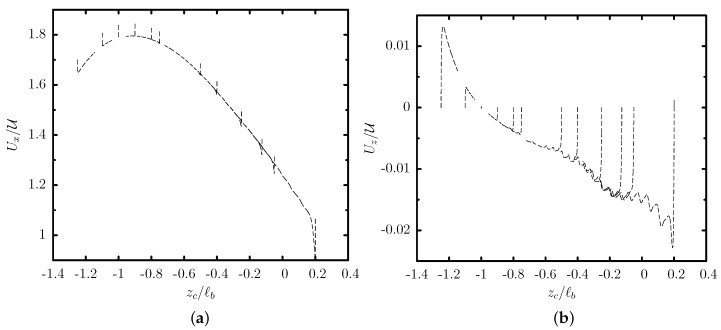
Evolution of (**a**) the translation velocity $U_{x}$ and (**b**) the migration velocity $U_{z}$, as a function of the centroid $z_{c}$, for a capsule with $a/\ell_{b}=0.55$, Ca=0.1 and different initial positions $z_{c}^{0}/\ell_{b}$ in the range [−1.25,0.2], flowing in a trapezoidal channel with height $\ell_{z}/\ell_{b}=2$ and base ratio $\ell_{a}/\ell_{b}=0.25$.

**Figure 3:**
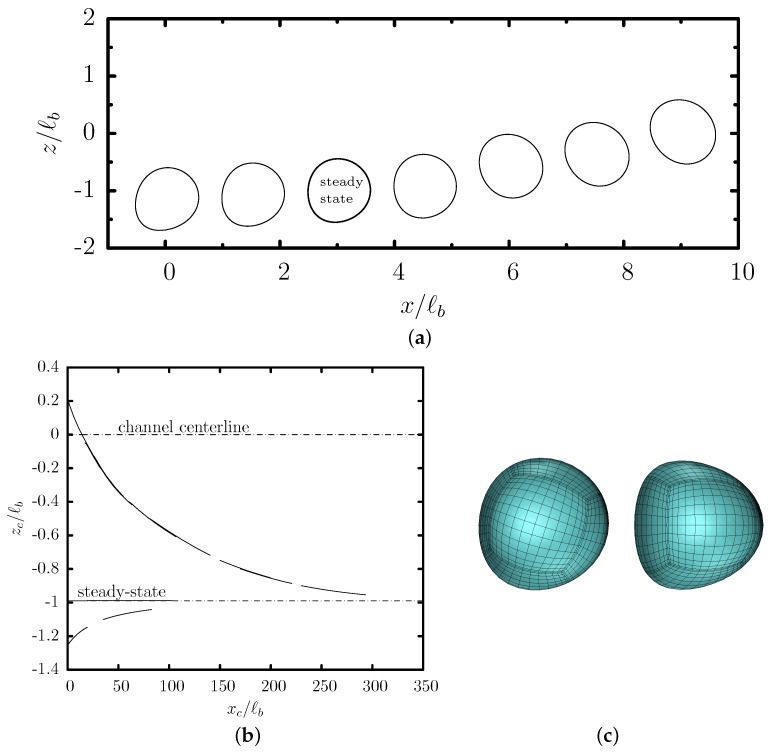
Evolution of a capsule with $a/\ell_{b}=0.55$ and Ca=0.1 flowing in a trapezoidal channel with height $\ell_{z}/\ell_{b}=2$ and base ratio $\ell_{a}/\ell_{b}=0.25$. (**a**) The quasi-steady front profile (i.e., intersection of the system surface with the plane y=0) at centroid $z_{c}/\ell_{b}=-1.25,-1.1,-0.99,-0.9,-0.5,-0.25,0.2$. These profiles were shifted along the *x*-axis to appear nearby but separated. (**b**) The capsule’s centroid $z_{c}$ versus its centroid $x_{c}$, derived from our computations for different initial positions $z_{c}^{0}/\ell_{b}$ in the range [−1.25,0.2]. (Note that our steady-state computations which cover the *x*-distance [0,100] were extrapolated to the entire *x*-range for optical reasons.) (**c**) Front and top views of the steady-state capsule shape.

Figure 2 shows the translation and migration velocity of this capsule as a function of its centroid $z_{c}$. After a short initial transient period (shown in this figure as vertical lines), the capsule shape and velocity become independent of its initial position, and thus the capsule follows a quasi-steady evolution which depends only on its centroid $z_{c}$. Owing to the cross-streamline migration associated with a non-zero velocity $U_{z}$ shown in Figure 2b, the capsule migrates towards the lower part of the trapezoidal channel at $z_{c}/\ell_{b}\approx -0.99$ where it obtains its steady-state shape and its translation velocity $U_{x}$ is nearly maximized as seen in Figure 2a. The quasi-steady front profiles of the capsule at different positions are shown in Figure 3a.

We emphasize that the cross-streamline migration is a slow process as it can be deduced from the capsule migration velocity $U_{z}$ which is at least two orders of magnitude smaller than its translation velocity $U_{x}$ as shown in Figure 2. In addition, the migration becomes much slower as the capsule approaches its steady-state location. Thus, experimental microfluidic systems should employ long channels for the capsules to reach steady-state, as shown in Figure 3b.

The migration velocity $U_{z}/U$ has a typical value of $10^{-2}$ far from its steady-state location, while it decreases to $10^{-5}$ at its steady-state location keeping this value for very long time periods there. We emphasize that owing to limitations of the numerical accuracy, the migration velocity cannot become exactly zero at the capsule’s steady-state location. However, we consider this very low value of the migration velocity $U_{z}/U\approx 10^{-5}$ as an indication that the capsule has reached its steady-state location in the trapezoidal channel.

We also note that a capsule placed further away from its steady-state location (e.g., near the channel centerline at $z_{c}^{0}/\ell_{b}=0$) undergoes a slow tank-treading on the y=0 plane which (along with the migration and thus deformation change) causes the small oscillations in the migration velocity $U_{z}$ observed in Figure 2b. The tank-treading speed is further reduced as the capsule approaches its steady-state location while the membrane tank-treads counter-clockwise on the xz-plane when it is above its steady-state location and clockwise when the capsule is below (or at) its steady-state location.

**Figure 4:**
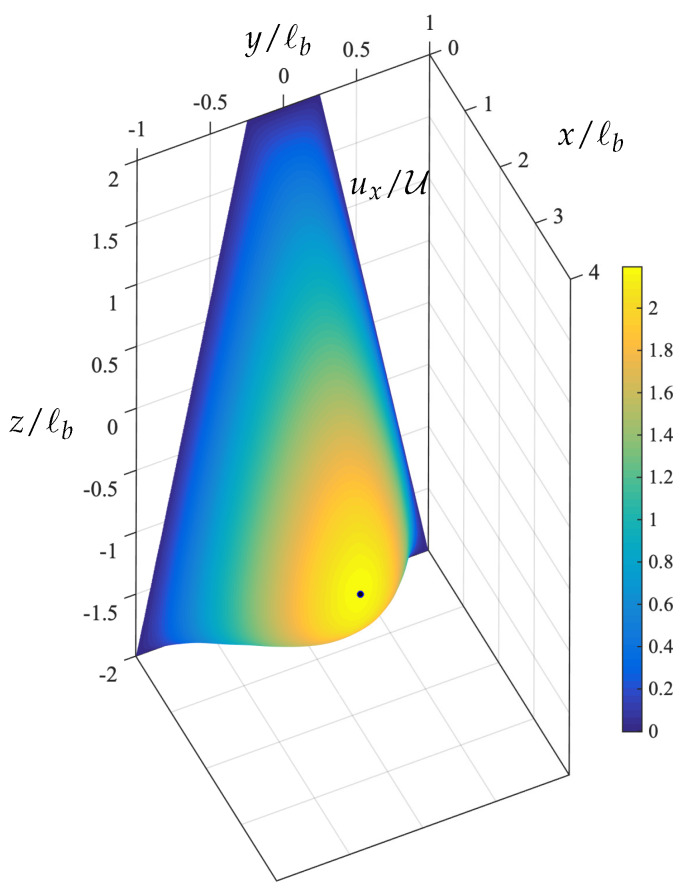
The velocity profile $u_{x}/U$ at the inlet of a trapezoidal channel with height $\ell_{z}/\ell_{b}=2$ and base ratio $\ell_{a}/\ell_{b}=0.25$. The maximum velocity is located at $z/\ell_{b}\approx -0.91$ as shown by the solid circle.

The steady-state location of the capsule in this trapezoidal channel is quite close to the location where the single-phase velocity of the surrounding fluid is maximized as shown in Figure 4. In essence, the capsule migration in the trapezoidal channel is similar to that in square channels where the capsule migrates at the channel centerline where the surrounding fluid velocity is maximized. However, owing to the asymmetry of the trapezoidal channel, the capsule’s steady-state shape is not symmetric along the *z*-axis as seen in Figure 3c and its location is slightly below the location of the maximum of the single-phase velocity.

**Figure 5:**
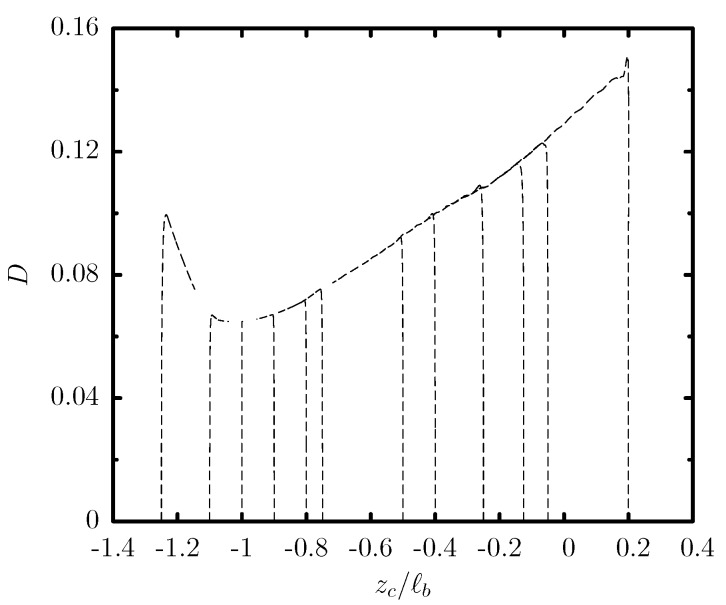
Evolution of the deformation *D* as a function of the centroid $z_{c}$, for a capsule with $a/\ell_{b}=0.55$, Ca=0.1 and different initial positions $z_{c}^{0}/\ell_{b}$ in the range [−1.25,0.2], flowing in a trapezoidal channel with $\ell_{z}/\ell_{b}=2$ and $\ell_{a}/\ell_{b}=0.25$. Note that D=(L−S)/(L+S), where *L* and *S* are the maximum and minimum distance of the capsule membrane from its centroid $x_{c}$.

To investigate further the quasi-steady deformation of the flowing capsule, we determined the maximum and minimum distance, *L* and *S*, respectively, of the capsule membrane from its centroid $x_{c}$. Note that these lengths occur at various orientations and thus represent the three-dimensional nature of the capsule deformation. Our investigation reveals that the steady-state capsule location is associated with a minimization of the length *L* and a maximization of the width *S*, and thus a minimization of the deformation D=(L−S)/(L+S) as shown in Figure 5.

It is of interest to note that the optimal lengths *L* and *S* require the complete knowledge of the three-dimensional capsule shape which is not available in experimental microfluidic systems, where the capsule can be observed only along the front view, and from the side view via prisms [49,50]. In essence, experimental systems can determine the capsule dimensions $L_{x}$, $L_{y}$ and $L_{z}$ along the three axes. Our computational results show that these lengths do not reveal the minimization of the capsule deformation at steady state, since these dimensions along the three co-ordinate axes are not, in general, representative of the three-dimensional capsule deformation in the trapezoidal channel.

### 3.2. Effects of the Channel Geometry and Capsule Size

**Figure 6:**
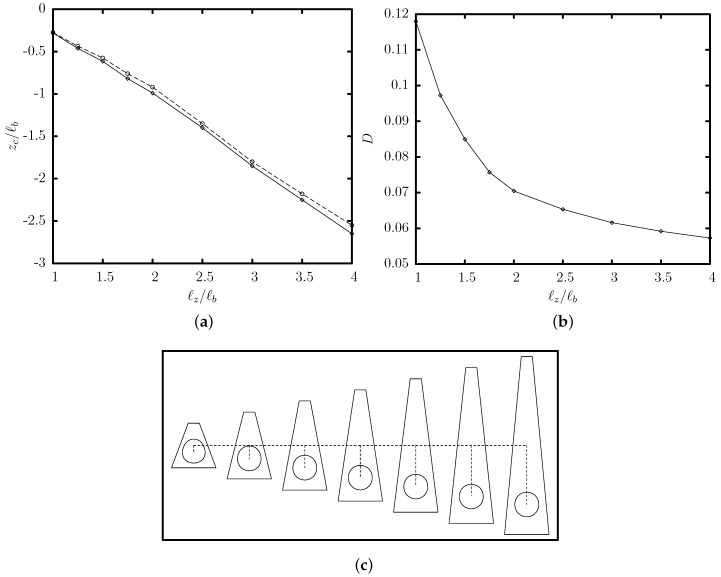
Steady-state variation with the channel height $\ell_{z}$ of (**a**) the centroid $z_{c}$ and (**b**) the deformation *D*, for a capsule with $a/\ell_{b}=0.55$ and Ca=0.1 flowing in a trapezoidal channel with base ratio $\ell_{a}/\ell_{b}=0.25$. In (**a**) also included is the location of the maximum inlet velocity (---). (**c**) The steady-state side profile (i.e., intersection of the system surface with the plane $x=x_{c}$) for channel height $\ell_{z}/\ell_{b}=1,1.5,2,2.5,3,3.5,4$.

We investigate now the effects of the channel’s height $\ell_{z}$ on the capsule’s steady-state properties. Figure 6 reveals that the capsule’s steady-state location is slightly lower than the location of the maximum of the single-phase channel velocity, in agreement with our earlier results. As the channel height $\ell_{z}$ increases for a given base ratio $\ell_{a}/\ell_{b}$, the steady-state location is shifted further away from the channel centerline. As shown in the capsule’s side profiles presented in Figure 6c, at small channel heights, the capsule causes a significant flow blocking and thus its interfacial deformation *D* is increased. In essence, tall trapezoidal channels can produce significant off-center capsule migration which may be useful in sorting processes.

**Figure 7:**
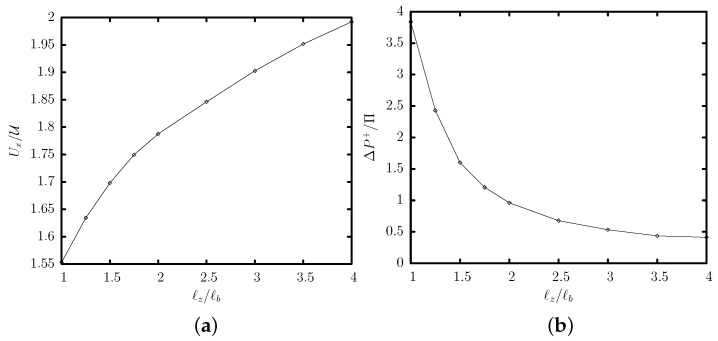
Steady-state variation with the channel height $\ell_{z}$ of (**a**) the translation velocity $U_{x}$ and (**b**) the additional pressure difference $\Delta P^{+}$, for a capsule with $a/\ell_{b}=0.55$ and Ca=0.1 flowing in a trapezoidal channel with base ratio $\ell_{a}/\ell_{b}=0.25$.

The increased flow blocking and proximity to the solid walls at small channel heights results in a significant decrease on the capsule’s translation velocity $U_{x}$ and a large increase on the additional pressure difference $\Delta P^{+}$ caused by the capsule presence, as shown in Figure 7. The variation of these two properties can be explained based on our earlier analysis for capsule motion in square or cylindrical channels [15]. In particular, the capsule velocity and the additional pressure difference scale roughly as

(10) $$\frac{U_{x}-U}{U}∼{\left(\frac{\Delta P^{+}}{\Pi }\right)}^{-1}∼\frac{h}{\ell_{z}}$$

where *h* is the distance (or gap) between the capsule surface and the solid walls of the channel. As the gap *h* is reduced at small channel heights, the capsule velocity $U_{x}$ is decreased while $\Delta P^{+}$ is increased in agreement with the computational findings of Figure 7.

**Figure 8:**
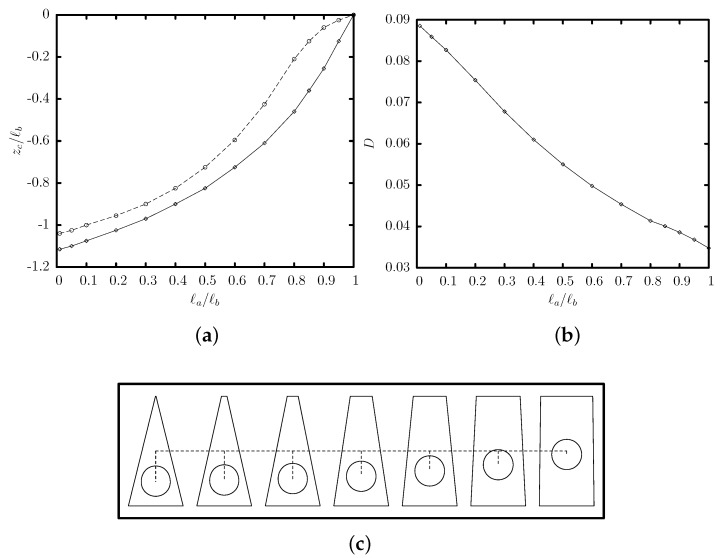
Steady-state variation with the base ratio $\ell_{a}/\ell_{b}$ of (**a**) the centroid $z_{c}$ and (**b**) the deformation *D*, for a capsule with $a/\ell_{b}=0.55$ and Ca=0.1 flowing in a trapezoidal channel with height $\ell_{z}/\ell_{b}=2$. In (**a**) also included is the location of the maximum inlet velocity $u_{x}$ (---). (**c**) The steady-state side profile (i.e., intersection of the system surface with the plane $x=x_{c}$) for channel’s base ratio $\ell_{a}/\ell_{b}=0.01,0.1,0.2,0.4,0.6.6,0.8,0.95$.

Figure 8 shows the effects of the channel’s base ratio $\ell_{a}/\ell_{b}$ on the capsule steady-state properties, for a trapezoidal channel with height $\ell_{z}/\ell_{b}=2$. Our computations cover the base-ratio range of (0,1], i.e., from a triangular channel with $\ell_{a}/\ell_{b}=0.01$ to a rectangular channel with $\ell_{a}/\ell_{b}=1$. Once again, the steady-state capsule location in a trapezoidal channel is slightly lower than the location of the maximum of the single-phase channel velocity. As seen from the side profiles presented in Figure 8c, when the channel’s base ratio is decreased, the higher flow blocking and proximity to the solid walls result in an increased interfacial deformation *D* (shown in Figure 8b) and additional pressure difference $\Delta P^{+}$ (not shown).

**Figure 9:**
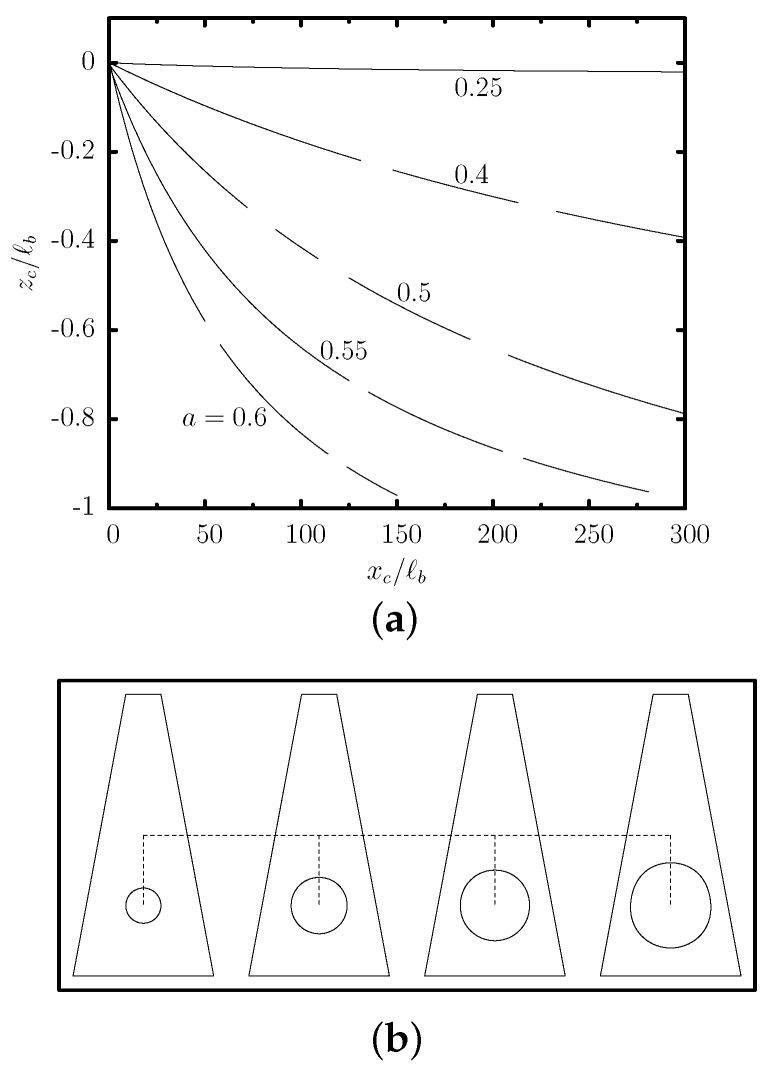
(**a**) Evolution of the centroid $z_{c}$ versus the centroid $x_{c}$, for a capsule with $a/\ell_{b}=0.25,0.4,0.5,0.55,0.6$ and Ca=0.1, flowing in a trapezoidal channel with $\ell_{z}/\ell_{b}=2$ and $\ell_{a}/\ell_{b}=0.25$. The curves were derived from our computations for different initial positions $z_{c}^{0}/\ell_{b}$ in the range [−1,0]. (**b**) The steady-state side profile for $a/\ell_{b}=0.25,0.4,0.5,0.6$.

We have also investigated the effects of the capsule size *a* for tall trapezoidal channels with small base ratios (i.e., $\ell_{z}/\ell_{b}=2$ and $\ell_{a}/\ell_{b}=0.25$) where significant off-center migration may occur. We considered capsules with size $a/\ell_{b}=0.25,0.4,0.5,0.55,0.6$ (i.e., smaller than the channel’s median width $\ell_{m}/\ell_{b}=0.625$), initially located along the centerline (y=z=0) of the trapezoidal channel. To compute efficiently their transient evolution towards steady state, for each capsule size *a* we considered several transient computations starting from different initial positions $z_{c}^{0}/\ell_{b}$ in the range [−1,0]. All capsules reach a steady-state location which is slightly lower than the location of the maximum of the single-phase channel velocity as we have seen earlier for capsule size $a/\ell_{b}=0.55$. Most important, as the capsule size is decreased, the lower capsule deformation and flow blocking in the channel (shown in Figure 9b) shift the capsule’s steady-state location nearer to the location of the maximum of the single-phase channel velocity. In particular, we found that for capsule size $a/\ell_{b}=0.6,0.55,0.5,0.4,0.25$, the steady-state location is $z_{c}^{\mathrm{SS}}/\ell_{b}\approx -1.0,-0.99,-0.97,-0.95,-0.93$.

Beyond the exact location at steady state, the transient dynamics of different capsules depend strongly on their size. Figure 9a shows the transient evolution of several capsules initially located along the centerline of a trapezoidal channel with $\ell_{z}/\ell_{b}=2$ and $\ell_{a}/\ell_{b}=0.25$. Small capsules (e.g., with size $a/\ell_{b}=0.25$) travel faster along the channel length but their migration is very slow. As the capsule size is increased, the capsule experiences stronger lift forces owing to the higher confinement by the channel walls [51,52], and thus migrates faster towards its steady-state position as seen in Figure 9a. For this specific channel geometry, the most efficient migration occurs for a capsule size $a/\ell_{b}=0.6$, i.e., close to the channel’s median width $\ell_{m}/\ell_{b}=0.625$.

Therefore, our investigation of the effects of the channel geometry and capsule size reveals that tall trapezoidal channels with low base ratios may be employed to produce significant off-center migration for large capsules compared to that for smaller capsules for a given channel length.

As a closure to our computational investigation, we emphasize that in this paper we have restricted our results to viscous capsules with viscosity ratio λ=5 for reasons of computational efficiency. However, we have also studied the migration of capsules with smaller viscosity ratio (namely, λ=0.1,1). We found that the behavior of these capsules is similar to that for λ=5. In particular, as the viscosity ratio decreases, the capsule migrates faster towards its steady-state location which is practically the same for all viscosity ratios. Therefore, the conclusions of our investigation are independent of the capsule’s viscosity ratio.

### 3.3. Design of A Capsule Sorting Microdevice

Based on our investigation of the capsule migration in trapezoidal channels, we propose a new microdevice for the sorting of artificial and physiological capsules based on their size *a*.

In particular, we propose utilizing tall trapezoidal microchannels with low base ratios, such as $\ell_{z}/\ell_{b}=2$ and $\ell_{a}/\ell_{b}=0.25$, which can be used to separate capsules with two different sizes *a* compared to the channel’s median width $\ell_{m}=\left(\ell_{a}+\ell_{b}\right)/2$. The capsules should enter the trapezoidal microchannel near its centerline (y=z=0), and tend to migrate towards their steady-state location with different migration velocities as seen in Figure 9a. Large capsules with size *a* close to the channel’s median width $\ell_{m}$ migrate much faster than smaller capsules. In essence, for the time or channel length needed for the large capsules to approach their steady-state location, small capsules remain practically near the channel centerline owing to their very small migration velocity.

**Figure 10:**
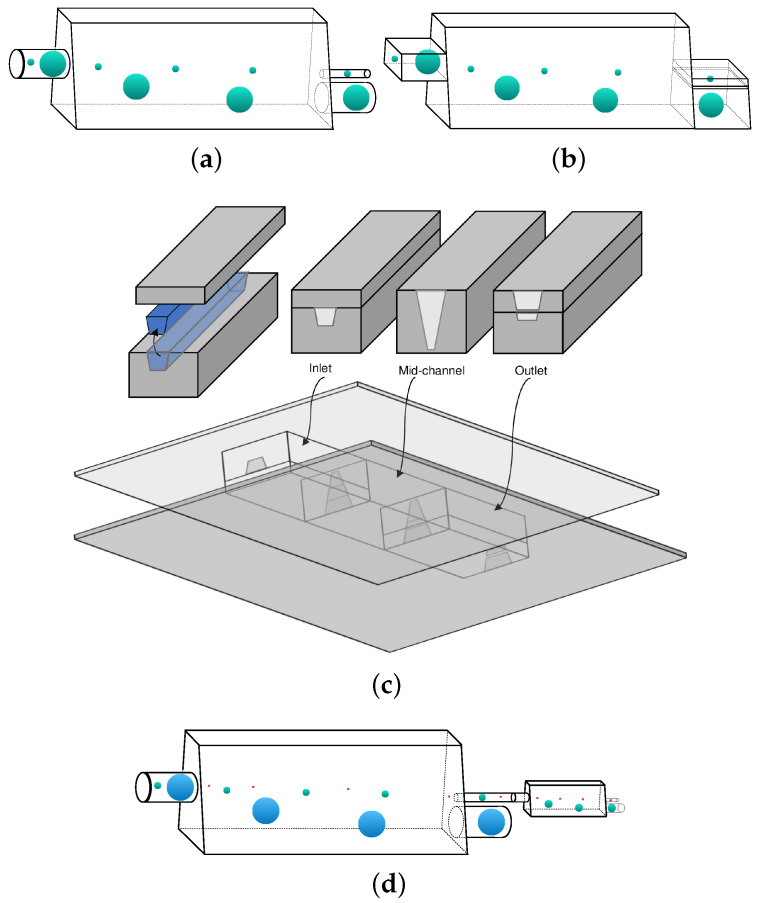
Illustration of the proposed sorting microdevice. (**a**) A micro-capillary consisting of the sorting trapezoidal channel with cylindrical inlet and outlets. (**b**) A microfluidic device with trapezoidal inlet and outlets. (**c**) Fabrication of the microfluidic device shown in (**b**). (**d**) A two-stage sorting device for the separation of capsules with three different sizes.

For a trapezoidal microchannel with $\ell_{z}/\ell_{b}=2$ and $\ell_{a}/\ell_{b}=0.25$, a large capsule with size $a/\ell_{b}=0.5$–0.6 requires a channel length $\ell_{x}/\ell_{b}\leq 500$ to migrate close to its steady-state location as seen in Figure 9a. For this channel length, a small capsule with size $a/\ell_{b}\leq 0.25$ remains practically at the channel centerline as shown in Figure 9a. The different capsules can thus be collected from the bottom and the center of the outlet of the trapezoidal channel as illustrated in Figure 10a,b. For a trapezoidal channel with a typical value of half-width $\ell_{b}=50\;\mu m$, the required channel length is $\ell_{x}=2.5\;\mathrm{cm}$ which can easily be constructed and monitored.

The proposed sorting microdevice can be readily produced via glass fabrication with cylindrical inlet and outlets as illustrated in Figure 10a. We emphasize that microdevices with different cross-sections, such as circular or rectangular, have been commonly produced via glass fabrication to study soft particles, e.g., for the generation of monodisperse double emulsions and the elasticity determination of soft gels [53,54,55].

The proposed sorting trapezoidal channel can also be fabricated as a microfluidic device with trapezoidal inlet and outlets as illustrated in Figure 10b. Several techniques including micromilling can be employed for the fabrication of trapezoidal channels made of plastic materials. To fabricate a trapezoidal duct, a rotating cutting tool equipped with a tapered endmill with a trapezoidal-like profile can be used to remove the bulk material as highlighted in blue in Figure 10c. The milled trapezoidal duct can then be bonded to a cover layer, resulting in completion of the channel inlet. Similarly, using endmills with appropriate profiles, the mid-channel and the outlet can be fabricated. The three pieces can be then bonded together and reinforced by the bottom and top cover layers as shown in Figure 10c. For more information on micromilling techniques we refer to the review article by Guckenberger et al. [56].

We emphasize that if the fluid stream collected from the centerline of the channel outlet contains capsules of different sizes (in our example, $a/\ell_{b}=0.25$ and $a/\ell_{b}\leq 0.10$), these capsules can also be sorted by utilizing in series another trapezoidal channel similar to the first one but with a smaller median width $\ell_{m}$ (in our example, comparable to $a/\ell_{b}=0.25$). A schematic example of a multi-stage system for separation of capsules with three different sizes is shown in Figure 10d.

The range of the required dimensionless parameters can be easily employed in our proposed experimental sorting microdevice without causing membrane rupture, e.g., see [16,49,57]. The requirement of low Reynolds number in the surrounding fluid is commonly achieved by employing high-viscosity surrounding fluids such as concentrated aqueous solutions of glycerin, Pale oils or silicon fluids [16,49,57]. (The Reynolds number of the capsule fluid is irrelevant since during the migration evolution the capsule translates as a solid-like particle with zero or very small inner circulation owing to the capsule’s very slow tank-treading.) For example, one can utilize trapezoidal micro-channels with half-width $\ell_{b}=50\;\mu m$ to sort micro-capsules made from a cross-linked ovalbumin membrane which have a shear modulus $G_{s}\approx 0.04\;N/m$ [16]. To achieve capillary numbers Ca=O(0.1), viscous stresses $\mu \;U=\mathrm{Ca}\;G_{s}=4\times 10^{-3}\;N/m$ need to be applied. Utilizing (nearly) 100% solutions of glycerin (with viscosity μ≈1Pas and density $\rho \approx 10^{3}\;{\mathrm{kg}/m}^{3}$), the average velocity should be U=4mm/s which is commonly employed in microchannel experiments with capsules without causing membrane rupture [16,49,57].

It is of interest to note that under these conditions, the external Reynolds number is Re=ρUℓb/μ=2×10−4. In addition, considering a density difference Δρ≤0.5ρ between the surrounding and inner liquids, and denoting as *g* the gravitational acceleration, the Bond number is $B_{d}=\Delta \rho \;g\;a^{2}/G_{s}\leq 4\times 10^{-4}$. We emphasize that our computations do not account for these two parameters since they assume that Reynolds and Bond numbers are identical zero. However, such low values of Reynolds and Bond numbers do not affect the deformation, migration velocity and steady-state position of a flowing capsule, e.g., [58,59].

Our proposed device can also separate biological cells from smaller impurities [60,61]. For example, hereditary spherocytosis is a blood disorder prevalent in people of Northern Europe ancestry which is caused by deficiencies of the spectrin network in the erythrocyte membrane, resulting in spherical-like erythrocytes [62,63]. This leads to a reduction of the membrane’s surface area and shearing modulus [63,64]. Owing to the small shearing modulus of these biological cells, $G_{s}=O\left(1\;\mu N/m\right)$, we can employ fluids with the viscosity of water including plasma, e.g., μ≈1mPas. For a cell size a≈3μm, we can utilize trapezoidal microchannels with half-width $\ell_{b}=5\;\mu m$. To achieve capillary numbers Ca=O(0.1), the average velocity should be U=0.1mm/s while the external Reynolds number is Re=5×10−4.

As a closure, we emphasize that a successful design of the proposed sorting device depends on the size of the channel’s median width $\ell_{m}$. In order to benefit from the wall effects and thus have faster migration, one may design a channel with a value $\ell_{m}$ close to the size of the larger capsule. In addition, by combination of different trapezoidal channels in a multi-stage series framework, sorting from a sample containing capsules of multiple sizes is feasible.

## 4. Conclusions

In the present paper we have investigated computationally the motion of an elastic spherical capsule in a trapezoidal microchannel at low and moderate flow rates under the Stokes regime. We note that the research interest in the last decades has been mainly focused on the motion of capsules in cylindrical or rectangular ducts. Therefore, the main goal of our work is to provide information on the capsule dynamics in channels with asymmetric cross-sections which may be desirable especially for capsule migration and sorting.

A capsule placed along the symmetry plane in an isosceles trapezoidal microchannel away from its steady-state location undergoes a cross-streamline migration which is accompanied with a slow tank-treading motion and a deformation decrease. These features are reduced as the capsule approaches its steady-state location near the location where the single-phase velocity of the surrounding fluid is maximized. Thus, the capsule migration in the trapezoidal channel is similar to that in square channels where the capsule migrates at the channel centerline where the surrounding fluid velocity is maximized. However, owing to the asymmetry of the trapezoidal channel, the steady-state capsule’s shape is also asymmetric while its membrane slowly tank-treads. Our investigation shows that the steady-state capsule location is associated with a minimization of the three-dimensional capsule deformation which cannot be revealed via single-view observation in experimental systems.

In addition, our investigation has revealed that tall trapezoidal channels with low base ratios may be employed to produce significant off-center migration for large capsules compared to that for smaller capsules for a given channel length, which may be useful in sorting processes.

Based on our investigation of the capsule migration in trapezoidal channels, we have proposed a new microdevice for the sorting of artificial and physiological capsules based on their size, by utilizing tall trapezoidal microchannels with low base ratios. The proposed sorting microdevice can be readily produced via glass fabrication or micromilling. The range of the employed dimensionless parameters can readily be used in our proposed experimental sorting microdevice without causing membrane rupture. In addition, the simple design of the proposed microdevice, paves the road for scale out and commercialization. Therefore, by combination of different trapezoidal channels in a multi-stage series framework, sorting from a sample containing capsules of multiple sizes is feasible.

Finally, we emphasize that different flowing particles (e.g., solid particles, droplets, capsules, microgels or vesicles) have in general different migration dynamics, and thus our study reveals the migration behavior of elastic capsules [22,25,27,28]. Therefore, we hope that our study provides motivation for more experiments with elastic capsules in channels with asymmetric cross-sections so that they are able to explore more the migration dynamics of these soft particles.
